# Commentary: The mediating effect of resilience between physical activity and mental health: a meta-analytic structural equation modeling approach

**DOI:** 10.3389/fpubh.2025.1599008

**Published:** 2025-06-23

**Authors:** Jiawei Zhao, Yufeng Wang, Chao Yang, Peiyang Guo

**Affiliations:** ^1^State Key Laboratory of Cognitive Neuroscience and Learning and IDG/McGovern Institute for Brain Research, Beijing Normal University, Beijing, China; ^2^Faculty of Psychology, Tianjin Normal University, Tianjin, China; ^3^Department of Psychology, Xinxiang Medical University, Xinxiang, China

**Keywords:** physical activity, resilience, mental health, meta-analytic structural equation model, bidirectional relationship

## Introduction

The complex relationship between physical activity (PA) and mental health (MH) has received substantial attention in recent years. Numerous studies have confirmed the beneficial effects of PA on MH (1, 2), including reductions in depressive and anxiety symptoms. However, the bidirectional nature of this relationship remains insufficiently explored. Longitudinal studies, such as the Whitehall II cohort study (3, 4), indicate persistent bidirectional associations between PA and MH. These studies not only demonstrate that PA positively influences MH, but also show that individuals with better MH are more likely to engage in PA. These findings align with the growing recognition of the bidirectional relationship between PA and cognitive function, as highlighted in the 2024 Lancet Commission report on dementia prevention, intervention, and care (5).

Lin et al. (6) innovatively employed a meta-analytic structural equation modeling (MASEM) approach to investigate the relationship between PA, resilience, and MH. They confirmed the positive impact of PA on MH and identified the mediating role of resilience. However, their analysis primarily focused on the unidirectional path from PA to MH. To comprehensively understand the complex interactions between these factors, it is necessary to further explore the bidirectional associations between PA, resilience, and MH. Therefore, the purpose of this commentary is to conduct a secondary analysis based on Lin et al.'s meta-analytic data (6) using the MASEM approach, aiming to further validate and extend their conclusions and provide more evidence for the bidirectional relationship between PA and MH.

## Methods

We utilized the coding data provided by Lin et al. (6) and performed statistical analyses using R software (version 4.4.2), employing the metaSEM (version 1.5.0) and lavaan (version 0.6-19) packages. First, we replicated the original analysis to verify the forward effect, whereby PA influences MH. Second, we examined the reverse effect, assessing the impact of MH on PA, with resilience as a mediating variable.

## Results

**Figure 1:**
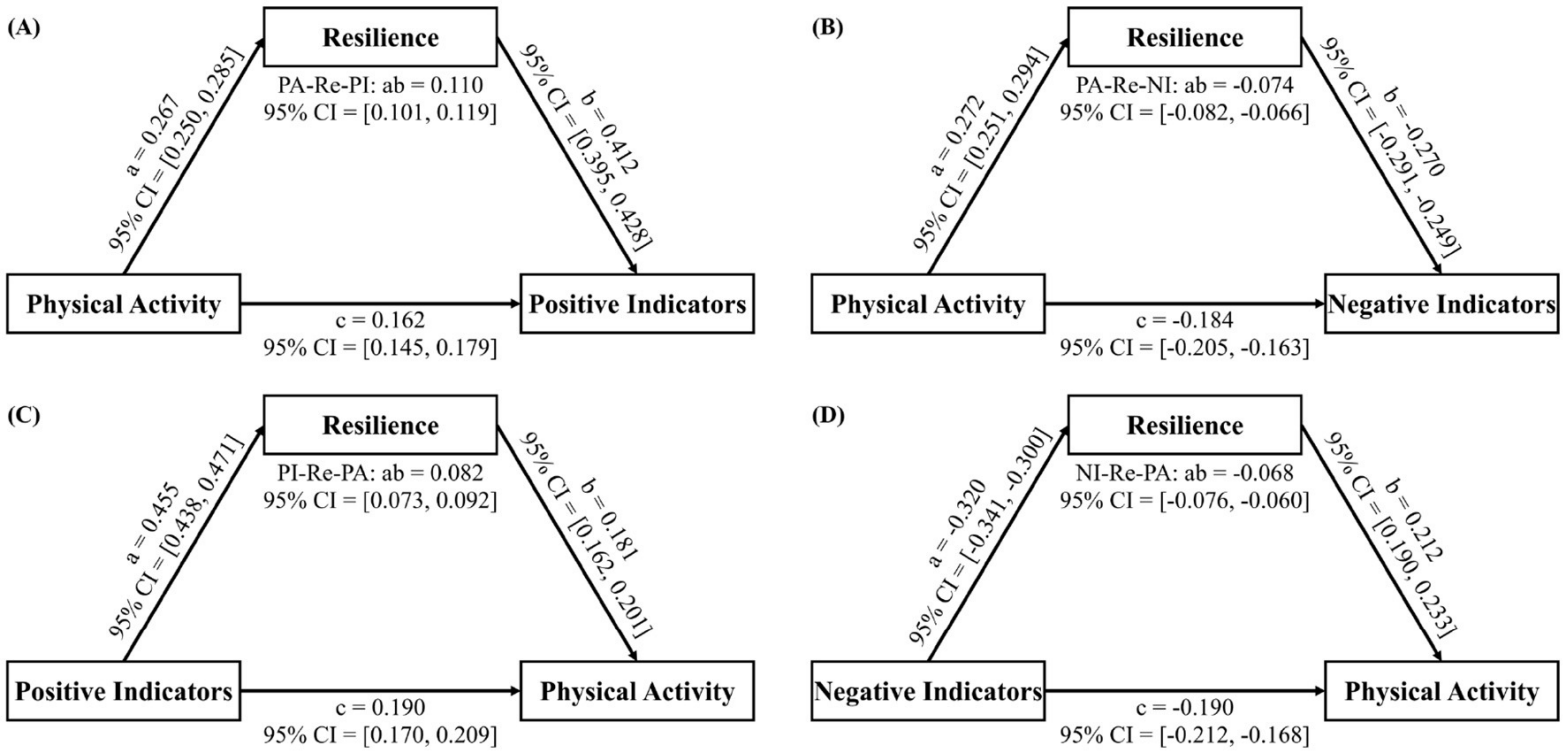
Bidirectional relationship between physical activity and mental health based on MASEM analysis. **(A, B)** Show the results of the forward analysis, where **(A)** illustrates the effect of PA on PI, and **(B)** illustrates the effect of PA on NI. **(C, D)** Show the results of the reverse analysis, where **(C)** illustrates the effect of PI on PA, and **(D)** illustrates the effect of NI on PA. PA, Physical Activity; Re, Resilience; PI, Positive Indicators; NI, Negative Indicators; MASEM, Meta-Analytic Structural Equation Model.

Our forward analysis replicated the findings of Lin et al. (6) (Figures 1A, B), confirming the significant direct effect of PA on both positive (c = 0.162, 95% CI = [0.145, 0.179]) and negative indicators of MH (c = −0.184, 95% CI = [−0.205, −0.163]), as well as the mediating role of resilience (positive indicators: ab = 0.110, 95% CI = [0.101, 0.119]; negative indicators: ab = −0.074, 95% CI = [−0.082, −0.066]).

The reverse analysis revealed that MH also significantly predicted PA levels (Figures 1C, D), with resilience partially mediating this relationship (positive indicators: c = 0.190, 95% CI = [0.170, 0.209], ab = 0.082, 95% CI = [0.073, 0.092]; negative indicators: c = −0.190, 95% CI = [-0.212, −0.168], ab = −0.068, 95% CI = [−0.076, −0.060]). The full code, data, and results are available on OSF (https://osf.io/3cmj7/?view_only=e60278f4a2754a5285aff0809d2d376f).

## Discussion

Our secondary analysis not only confirmed the reliability of Lin et al.'s conclusions (6) but also extended their findings by demonstrating the bidirectional relationship between PA and MH. The forward effect suggests that engaging in PA fosters resilience, which, in turn, enhances MH. Conversely, the reverse effect indicates that individuals with better MH are more likely to maintain regular PA, partially due to their stronger resilience. As shown in the Whitehall II study, depressive symptoms and anxiety can significantly reduce PA adherence, emphasizing the important role of MH in motivating PA engagement (3). These findings support a dynamic and reciprocal feedback loop between PA, resilience, and MH, offering new insights into the biopsychosocial model of health (7) and expanding it to incorporate resilience-specific frameworks such as the Connor-Davidson Resilience Scale (8).

The bidirectional relationship between PA and MH highlights the need for integrated intervention strategies that simultaneously target these factors and resilience. For sub-healthy populations, focusing on increasing PA levels and fostering resilience may synergistically enhance overall health. For individuals diagnosed with mental disorders, combining pharmacotherapy with appropriate exercise interventions and resilience training can optimize treatment outcomes and reduce the risk of relapse (9).

From a theoretical perspective, recognizing the bidirectional relationship between PA and MH advances our understanding of the intricate interplay between modifiable risk factors and psychological wellbeing. This knowledge can inform the development of targeted, multi-level interventions that acknowledge the reciprocal nature of these associations. Practically, our findings highlight the importance of incorporating PA and resilience-building strategies into MH promotion and treatment frameworks. Additionally, they provide a new perspective on exploring how MH, as a reverse driving factor, can further improve an individual's MH levels when promoting PA.

However, some limitations should be acknowledged, such as the relatively small number of included studies and the lack of heterogeneity tests across participant characteristics and measurement tools. Future research should aim to expand the sample size, explore potential moderators, and incorporate additional mediating factors to establish a more comprehensive, multi-level model of the PA-MH relationship.
